# Patient-derived organoids of lung cancer based on organoids-on-a-chip: enhancing clinical and translational applications

**DOI:** 10.3389/fbioe.2023.1205157

**Published:** 2023-05-26

**Authors:** Xiao Zeng, Qiong Ma, Xue-Ke Li, Li-Ting You, Jia Li, Xi Fu, Feng-Ming You, Yi-Feng Ren

**Affiliations:** ^1^ Hospital of Chengdu University of Traditional Chinese Medicine, Chengdu, Sichuan, China; ^2^ Cancer Institute, Chengdu University of Traditional Chinese Medicine, Chengdu, Sichuan, China; ^3^ Department of Laboratory Medicine, West China Hospital, Sichuan University, Chengdu, Sichuan, China

**Keywords:** lung cancer, patient-derived organoids, drug screening, microfluidic chip, organoids-on-a-chip

## Abstract

Lung cancer is one of the most common malignant tumors worldwide, with high morbidity and mortality due to significant individual characteristics and genetic heterogeneity. Personalized treatment is necessary to improve the overall survival rate of the patients. In recent years, the development of patient-derived organoids (PDOs) enables lung cancer diseases to be simulated in the real world, and closely reflects the pathophysiological characteristics of natural tumor occurrence and metastasis, highlighting their great potential in biomedical applications, translational medicine, and personalized treatment. However, the inherent defects of traditional organoids, such as poor stability, the tumor microenvironment with simple components and low throughput, limit their further clinical transformation and applications. In this review, we summarized the developments and applications of lung cancer PDOs and discussed the limitations of traditional PDOs in clinical transformation. Herein, we looked into the future and proposed that organoids-on-a-chip based on microfluidic technology are advantageous for personalized drug screening. In addition, combined with recent advances in lung cancer research, we explored the translational value and future development direction of organoids-on-a-chip in the precision treatment of lung cancer.

## Graphical Abstract

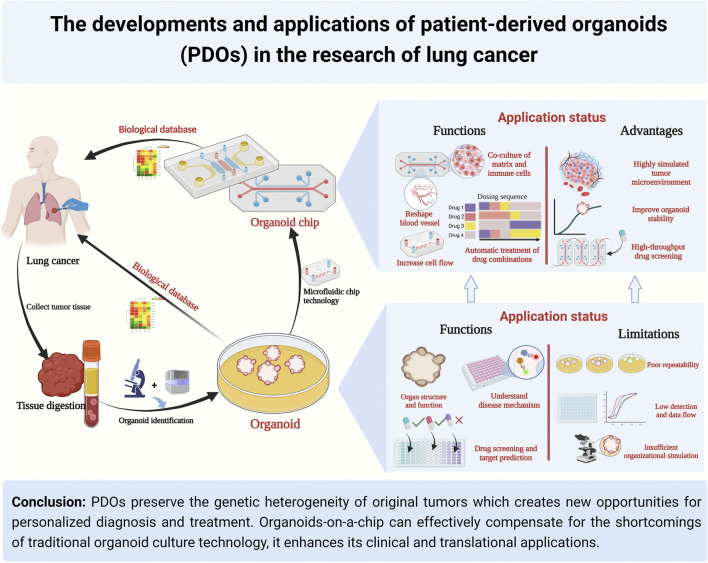

## 1 Introduction

Lung cancer was the second most incident malignant tumor and the leading cause of cancer-related death in 2020 (Sung et al., 2021). Although targeted therapy and immunotherapy have accurately treated lung cancer and effectively improved the remission and disease control rates, prolonging the overall and progression-free survival of patients (Chen et al., 2021; Liang et al., 2021; Kolesar et al., 2022), this cancer still has one of the highest mortality rates. Some main reasons for the poor prognosis of lung cancer are the genetic differences between patients, the inherent phenotypic and genetic heterogeneity of tumor tissues, and the difficulty of explaining the internal causes of tumorigenesis and developing individualized treatments. Therefore, the 5-year survival rate of patients with lung cancer remains <20% (Boumahdi and de Sauvage, 2020; Cao et al., 2020). Finding good preclinical models, individualized drug screening, and new drug research and development are of great significance for accurately treating lung cancer.

Current preclinical models of lung cancer can be categorized into *in vitro* and *in vivo* models, but while the widely used *in vitro* cell line model is simple and cost-effective, it overlooks the tumor heterogeneity and lacks the complex cell structure of the tumor microenvironment (TME), making it challenging to guide individualized clinical drug screening and proper treatment (Kodack et al., 2017; Noguchi et al., 2022), whereas *in vivo* models like Genetically Engineered Mouse (GEM) and Patient-Derived Xenograf (PDX) provide a TME but are costly, time-consuming, and their consistency with human TME remains to be explored (Hui and Chen, 2015; Morgan et al., 2017), but with the emergence of three-dimensional culture technology (Tuveson and Clevers, 2019; Parekh et al., 2022), the patient-derived organoids (PDOs) model has become an attractive option due to its short culture cycle, cost-effectiveness, and potential for personalized drug screening. Also, PDOs, along with their extended biobanks, retain the real genetic information of the tumor tissues of patients. PDOs play an important role in the development of disease models and drug screening tests. These characteristics that make them a highly suitable preclinical model for quickly translating significant scientific discoveries into personalized therapies.

However, traditional culture methods of PDOs have technical bottlenecks, such as poor stability, difficulty in high-throughput drug screening, lack of a co-culture system of vascular and tumor-infiltrating immune cells, and lack of stimulation of the tissue environment *in vivo* (Hu et al., 2021; Hofer and Lutolf, 2021; Picollet-D’hahan et al., 2017). To overcome these problems, researchers from the manufacturing technology and material science field have strengthened multidisciplinary exchanges and cooperation, broken down technical barriers, and innovatively put forward technical means such as gas-liquid interface, 3D printing, and organoids-on-a-chip (Qu et al., 2021; Xia et al., 2021; Xu et al., 2021), making the growth and development of organoids more accurate and controllable, the structure more simulated, and the organoid model continually developing towards anthropomorphism, automation, and integration. In 2021, the US Food and Drug Administration (FDA) indicated a positive attitude towards organoids-on-a-chip in new drug research and development and precision treatment to gradually reduce or replace animal models by establishing a standardized model platform and using organoids-on-a-chip to fill the gaps in various diseases and physiological models. In August 2022, the FDA first approved a new drug (NCT104658472) for clinical trials based solely on preclinical efficacy data obtained from organoids-on-a-chip studies.

This review discusses the application status and current challenges of PDOs culture systems, summarizes the advantages and characteristics of organoids-on-a-chip as a personalized drug screening model, and combines the latest progress in lung cancer research to explore the transformation value and future development direction of organoids-on-a-chip for the accurate treatment of lung cancer.

## 2 PDOs culture technique and applications in lung cancer

### 2.1 Definition and characteristics of PDOs

PDOs are three-dimensional structures that are derived from the cells of patients and are cultured *in vitro* to replicate the cellular and molecular characteristics of the original tissue. PDOs are typically composed of a mixture of different cell types that are organized in a manner that resembles the architecture of the original tissue. PDOs have several key characteristics include: 1) Genetic and phenotypic fidelity: PDOs retain the genetic and phenotypic characteristics of the original tissue from which they were derived, making them valuable for studying disease mechanisms and drug responses. 2) Heterogeneity: PDOs contain a mixture of different cell types, similar to the original tissue, which allows for the study of complex cell interactions and disease heterogeneity. 3) Self-renewal and differentiation: PDOs have the ability to self-renew and differentiate into different cell types, providing a platform for studying tissue development and regeneration. 4) Response to treatment: PDOs can be used to test the efficacy of different drugs and treatment strategies, providing a personalized approach to drug discovery and clinical decision-making. Overall, PDOs provide a powerful tool for studying disease mechanisms, drug discovery, disease modeling and personalized medicine, with the potential to revolutionize the field of precision medicine.

### 2.2 Advantages of using PDOs for lung cancer research

#### 2.2.1 Personalized disease modeling

Lung cancer is a complex and heterogeneous disease that can affect tumor behavior, response to treatment, and patient outcomes (Sacher et al., 2016) (Hirsch et al., 2017). There are two main types of lung cancer: non-small cell lung cancer (NSCLC) and small cell lung cancer (SCLC). NSCLC is the most common type, accounting for around 85% of all lung cancer cases. NSCLC is further divided into three subtypes: adenocarcinoma, squamous cell carcinoma, and large cell carcinoma. Each of these subtypes has unique characteristics, genetic alterations, and responses to treatment (Hirsch et al., 2017), presenting considerable challenges to the use of precision medicine

**FIGURE 1:**
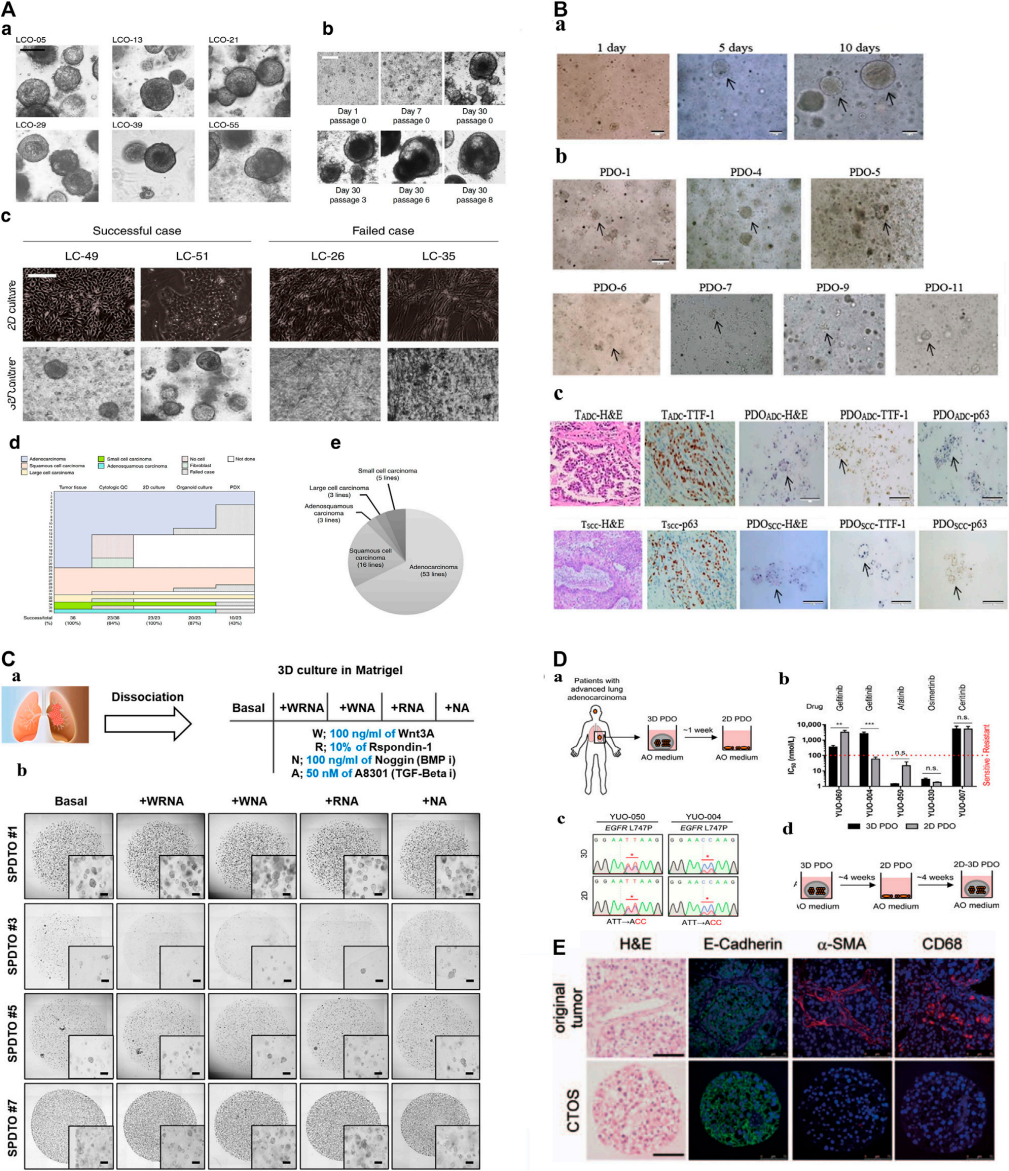
The construction of biobanks containing complex biological characteristics of several kinds of tumor subtypes by utilizing different methods. **(A)**: a. Bright-field microscopy images of LCOs. b. Representative images of long-term cultured LCOs. c. Representative images of successful and failed 2D and 3D cultures derived from lung cancers with different tissue composition. d. The establishment rate of each cancer models according to lung cancer subtypes. e. The subtypes of established 80 LCOs for the lung cancer biobank (Kim et al., 2019). “Adapted with permission from (Kim et al., 2019). Copyright The Author(s) 2019.” **(B):** PDOs of NSCLC recapitulate the parental tumor. a. The PDO phenotype was observed with a bright field microscope. b. Morphological changes of PDOs. c. HE-stained and IHC images of PDOs and their parental tumor tissues (Chen et al., 2020). “Adapted with permission from (Chen et al., 2020). Copyright 2020 American Chemical Society.” **(C)**: Establishment of tumor organoids derived from SCLC. a. Tumor tissues were obtained from SCLC patients and cultured under various conditions to form organoids. b. Brightfield images of SPDTO formation in short-term culture under five different culture conditions (Choo et al., 2021). “Adapted with permission from (Choi S. Y. et al., 2021). Copyright 2021 by the authors.” **(D):** Procedure for generating 2D PDOs. a. Procedure for generating 2D PDOs. b. Comparison of IC50 values to each TKI between 3D and 2D PDOs. c. DNA chromatograms showing EGFR L747P mutation. d. Scheme for model switching (Kim S. Y. et al., 2021). “Adapted with permission from (Kim S. Y. et al., 2021). Copyright 2021 The author.” **(E):** Characterization of CTOSs from patients’ lung tumors with H&E staining and immunohistochemistry of E-cadherin, α-SMAand CD68 (Endo et al., 2013). “Adapted with permission from (Endo et al., 2013). Copyright 2013 International Association for the Study of Lung Cancer.“Abbreviations: PDOs, patient-derived organoids; LCOs, lung cancer organoids; NSCLC, non-small cell lung cancer; SCLC, small cell lung cancer; SPDTO, SCLC patient-derived tumor organoid; TKI, tyrosine kinase inhibitor; CTOSs, cancer tissue-originated spheroid; EGFR, epidermal growth factor receptor.

PDOs as a good preclinical model, retain the heterogeneity of the original tumor tissue which allows for personalized disease modeling, opening up a new direction for personalized and precision treatment of lung cancer. For example, Kim et al. (2019) published lung cancer PDOs containing five lung cancer subtypes PDOs (adenocarcinoma, squamous cell carcinoma, adenosquamous carcinoma, large cell carcinoma, and small cell carcinoma), and five normal bronchial organoids (NBOs) of 80 patients, which covers more than 95% of lung cancer patients, providing tumor organoids cultures representing the complexity of different tumor subtypes and inter-patient heterogeneity (Figure 1A; Table 1). Subsequently, Li Z. et al. (2020), Banda et al. (2020), and Chen et al. (2020) have successfully established the living biological bank of PDOs in different subtypes of lung cancer and normal lungs (Figure 1B; Table 1). Those suggest that PDOs show a high degree of genotypic and phenotypic consistency with the original clinical specimens, which takes into account the unique genetic and molecular characteristics of each patient’s tumor. This can enable the development of personalized medicine and improve patient outcomes.

**TABLE 1:** Application status of lung cancer organoids derived from patients.

Sample source	Success rate	Applications and methodologies	Conclusion	Biobank	References
NSCLC (surgical specimens, pleural effusions)	80%	Drug sensitivity assay: PDOs were exposed to erlotinib for 7 days, viability was evaluated based on size.	The first three-dimensional culture system for lung cancer patients	-	Endo et al. (2013)
lung adenocarcinomas	-	Drug predicting: at Days 8 and 15 were used to analyze spheroid count, average size, and total area	PDOs enable to assess outgrowth of mutant subpopulation	-	Banda et al. (2020)
SCLC	80%	Drug test: withdrew the treatment after 10 days, and monitoring viability for a further 4 weeks.	Long-term expansion experiments were carried out by adding WNT3A or R-spind1	-	Choi et al. (2021a)
lung adenocarcinoma (malignant effusion)	83%	Targeted therapies test and drug screening: cell viability assay in 15days	The drug response of PDOs is related to clinical results	n = 100	Kim et al. (2021b)
SCLC and NSCLC	44%	Drug sensitivity testing: after 10–14 days culturing, added drugs every 3 days, 6 days for cell viability	PDOs can be used to predict patient-specific drug responses	n = 80	Kim et al. (2019)
NSCLC	33%	Co-culture of peripheral blood lymphocytes and tumor organoids	Expanding immune cells is difficult	-	Dijkstra et al. (2018)
NSCLC	-	Drug sensitivity testing: after 7–10 days for drug treatment, Cell viability was assessed	A reliable model for predicting the response to medical treatment.	-	Chen et al. (2020)
NSCLC	88%	Drug testing: PDOs was exposed to drug for 3 days and for cell viability	PDOs for drug testing and biomarker validation is demonstrated.	-	Shi et al. (2020)
		*In vivo* organoid implantations			
NSCLC	-	Drug treating with natural compounds: PDOs were exposed to drug for 6 days, and measuring the cell viability	The response of lung cancer PDOs to various natural compounds was studied	n = 10	Li et al. (2020b)

Abbreviations: PDOs, patient-derived organoids; NSCLC, non-small cell lung cancer; SCLC, small cell lung cancer; NOD/SCID, Non-obese diabetes mellitus/severe combined immunodeficiency; -, not mentioned.

#### 2.2.2 Drug sensitivity test and drug development

Selecting PDOs for detecting drug sensitivity and guiding individualized clinical treatment of lung cancer is of great significance, as current drugs for treatable gene mutations may not meet patient needs due to genetic differences. Inappropriate drug selection can cause treatment failure. (Xia et al., 2021; Sun et al., 2022). The latest study published in Cell Reports Medicine used Fifty-four PDOs derived from 36 patient tumors to test the sensitivity of tumors to osimertinib, chemotherapy, dual-targeted therapy, and other targeted therapy, with overall 84.0% sensitivity, 82.8% specificity, and 83.3% accuracy (Wang et al., 2023). and found that the response to treatment varied depending on the genetic characteristics of the tumor. Moreover, Shi et al. (Shi et al., 2020) (Table 1). utilized PDOs-based drug testing revealed that the combination of FGFR and MEK inhibitors showed better treatment effects than a single FGFR inhibitor in FGFR1 amplified lung adenocarcinoma, this suggested that PDOs can be used to test the sensitivity of individual tumors to various treatments and to develop personalized treatment strategies for patients based on their specific tumor characteristics, highlighting the important translational nature of PDOs-based personalized medicine. Similarly, Choi S. Y. et al. (2021) (Figure 1C; Table 1), Kim S. Y. et al. (2021) (Figure 1D; Table 1), Chen et al. (2020) established SCLC/NSCLC PDOs (*n* = 10,77,7) and studyed their responses to drugs, all arrived at the same conclusion that the drug test results are in good agreement with clinical outcome, these researches will not be elaborated in detail here.

In addition to drug sensitivity testing, PDOs can also be used for drug development by enabling the screening of large libraries of compounds for potential anti-cancer activity. A study published in 2020 used PDOs derived from SCLC patient tumors to develope a novel CDK7 inhibitor, YPN-005, which showed potent anticancer effects compared to the CDK7 inhibitor THZ1 (Choi Y. J. et al., 2021). Meanwhile, the response of lung cancer PDOs to various natural compounds for potential anti-cancer activity was studied, and the anticancer activity of various natural compounds, such as chelerythrine chloride, cantharidin, and hamming, which demonstrated a positive response. Similarly, lung cancer PDOs verify the anticancer activity of MFF D) 8–11 peptide mimic, and CKD9 inhibitors SNS032, LY2857785, AZD4573 (Seo et al., 2019) (Padmanabhan et al., 2021).

Overall, drug sensitivity testing and drug development using PDOs are promising approaches for developing personalized therapies for lung cancer patients, and have the potential to avoid ineffective therapies for patients to prevent side effects, time consumption, and resource expenditure, it’s expected to improve patient outcomes.

#### 2.2.3 Biomarker discovery

Clear biomarkers are important for the accurate diagnosis and treatment of tumors. PDOs have been widely used for biomarker discovery in different cancer research. Such as prostate cancer, liver cancer, and breast cancer, to anti-tumor drugs (Choi et al., 2015; Riahi et al., 2016; Heninger et al., 2021) to identify candidate biomarkers of drug sensitivity and resistance. For example, researchers can rapidly detect fluctuations in prostate cancer-related epigenetic biomarkers (RASSF1, APC, and RARb gene hypermethylation) before and after drug administration. Similarly, several studies have used lung cancer PDOs to identify biomarkers associated with drug sensitivity. For example, Shi et al. (2020) established NSCLC organoids and utilized transcriptomics analysis, revealing that the combination of FGFR and MEK inhibitors showed better treatment effects than a single FGFR inhibitor in FGFR1 amplified LUSC, which supports researchers’ earlier contention that PDOs can retain the targeted therapy sensitivity of its source tumor tissue. Furthermore, PDOs can also be used as discovery tools for novel biomarker and combination therapy approaches. The researchers (Bhaumik et al., 2020) also constructed lung cancer PDOs to detect the expression of multiple critical biomarkers on a single slide, demonstrating that the expression status and spatial arrangement of multiple disease-relevant biomarkers can be studied in a preclinical nontumor microenvironment setting. Overall, PDOs have proven to be a valuable tool for biomarker discovery in lung cancer research, with the potential to improve diagnosis and treatment options for patients.

### 2.3 Methodologies utilized for PDOs culture systems

PDOs culture systems refer to the process of generating of organoids from patient-derived tissue, characterization and testing the organoids for drug sensitivity and response et al., which for utilizing them in research and clinical applications. PDOs culture methodology has gained widespread popularity in the past few years (Basak et al., 2009; Mancini et al., 2011). Hydrogels composed of Matrigel-based culture is currently the most widely used method for generating PDOs (Pamarthy and Sabaawy, 2021), we will discuss this traditional and popular method here.

The first three-dimensional culture system for lung cancer patients was established by Endo et al. (Endo et al., 2013). Fresh primary human lung cancer tissues were sectioned and digested (Figure 1E; Table 1). The precipitated cells were cultured in Matrigel using a serum-free human embryonic stem cell medium or DMEM/F12, BSA, and Neuregulin-1. The success rate was 80%, and the sensitivity of the targeted drug erlotinib corresponded to the clinical results by using image analysis in 7 days. However, this study did not further compare the molecular characteristics of the model with those of the primary tumor, nor did it report the time to cell passage cultivation or amplification. With the continuous development of organoid field, lung cancer PDOs culture system has been used by more and more researchers. Such as, Choi S. Y. et al. (2021), Wang et al. (2023) have concentrated on developing various types of personalized disease models for lung cancer by employing next-generation sequencing (NGS) techniques. These studies confirmed the authenticity of the organoids by comparing their genomic profiling with that of the original tissue, characterizing the organoids to ensure their accurate representation of the patient’s tumor, and subsequently determining the drug sensitivity and response, which is complex to manipulate manually. For instance, to investigate the effect of PDOs, a manual execution approach was utilized, where PDOs were treated with multiple drugs in triplicate every 3 days, and their viability was assessed at specific time points using a 96/384-well plate format. However, such a method can be laborious and time-consuming. (Kim et al., 2019; Sachs et al., 2019; Kim S. Y. et al., 2021).

However, what is noteworthy is that there are several crucial factors to the use of precision medicine.

(1) Variable success rate: recent studies have reported a success rate ranging from 7% to 88% (Sachs et al., 2019) (Lee et al., 2021), the variability in success rates across different studies suggests that the culture process may not always be reproducible. The irregular manual manipulation involved in these laborious laboratory procedures could be a contributing factor to this variability.
(2) Low-effectiveness: Manually operating the traditional organoid culture systems is complicated (Bhaumik et al., 2020), especially in investigated expression patterns of key biomarkers or testing drug sensitivity and response at indicated time points to each well. Meanwhile, it is generally recognized that PDOs successfully cultivated during passage 3 were regarded as success in organoid formation (Kim et al., 2019). The time required to establish tumor organoids and complete the test is approximately 4–6 weeks. Therefore, the effectiveness of the process is limited.
(3) A low level of biofidelity: the advantages of PDOs biofidelity are that they can better replicate the heterogeneity, making them more representative of the patient’s cancer. This allows for more accurate drug screening and personalized treatment selection. However, they may not fully capture the complexity of the original tumor (Neal et al., 2018) (Most PDOs models have tumor cells but rarely key microenvironmental factors: immune and stromal cells and cytokines), which may limit their widespread use. Such as, only chemotherapy, targeted therapy but no immunotherapy and Anti-vascularization therapy (Yuki et al., 2020).
(4) Organoid biobank: An organoid biobank is a collection of organoids that have been generated and stored for research or clinical purposes. Organoid biobanks are becoming increasingly important for advancing precision medicine and accelerating drug discovery, as they provide a valuable resource for researchers and clinicians to study disease and develop targeted therapies. One of the key factors of the organoid biobank will be the efficient reconstitution of cryopreserved organoids. However, researchers have carefully observed the effects of centrifugation, freezing, and other processes on the stability of PDOs and found that these routine steps may cause cell differences and reduce their sensitivity to drugs (Yu et al., 2019; Liu et al., 2021). Overall, the traditional PDOs models still have some challenges, which bring uncertain factors to the future clinical application of PDOs. Thus, how to improve and optimize the PDOs model is crucial for the further clinical transformation and application.

## 3 The preclinical model of organoids-on-a-chip based on microfluidic technology

### 3.1 Definition and characteristics of organoids-on-a-chip

A review (Park et al., 2019) published in Science journal first proposed the concept of organoids-on-a-chip (the integration of organoids with microfluidic technology to create a platform that mimics the *in vivo* microenvironment of organs *in vitro*), also known as microphysiological systems (MPS), which emulating perfusion, mechanical and other parameters crucial for tissue and organ physiology. This technology combines the human cellular and tissue fidelity found in organoids and the environmental control of microfluidics chips leading to a better, more accurate technology, enables the study of organoids in a more physiologically relevant and controlled environment, allowing for the investigation of complex biological processes such as drug testing, and disease modeling. The term “microfluidics chips” is sometimes used to refer to the devices of organoids-on-a-chip that achieve precise control over the microenvironment surrounding cells in MPS, microfluidics chips and organoids-on-a-chips are related in microfluidics technologies but distinct in studying organ-level biology *in vitro*. Both microphysiological systems come in different sizes and shapes for precise control of fluid flow, they contain small and hollow channels seeded with living cells, but the former typically rely on flat, two-dimensional cell cultures. However, organoids-on-a-chip generally consist of microfluidic channels that provide: 1) high anthropomorphism, such as 3D co-culture cells, a continuous flow of nutrients, gases, shear, stress and tensile forces et al. 2) Microenvironmental control of organoids for administering drugs to organoids, such as control different Drug concentration gradient within a single microfluidic device. 3) Integration of biosensing, when test drugs to the organoids, allowing for real-time monitoring and analysis of their behavior by incorporating biosensing elements into culture platforms (Sontheimer-Phelps et al., 2019) (Park et al., 2019).

### 3.2 Advantages of using organoids-on-a-chip based on microfluidic technology for cancer research

#### 3.2.1 Improve the stability of organoids

It is important to note that the success rate of lung cancer PDOs culture can vary widely depending on the specific methodology used and the characteristics of the individual patient sample, but Endo et al. (Endo et al., 2013) and Wang et al. (Wang et al., 2023) both proposed that the difference may be partly because of the relatively poor culture conditions, such as necessary nutrient supply, biochemical microenvironment gradients mechanical forces (shear or stretch force). Schuster et al. also propose that organoids from the same patient are not identical in shape, size, and viability due to inconsistent manual manipulation during tedious laboratory procedures (Schuster et al., 2020).

The development of microfluidic technology has improved the consistency and stability of the PDOs model. Previous studies have confirmed that microfluidic technology can process or manipulate micro fluids (Garreta et al., 2021). The microfluidic chips produce various autocrine or paracrine cytokines by regulating shear stress, interstitial fluid flow, and cell density and forming different extracellular matrix characteristics. The matrix characteristics potentially affect physiological conditions such as organoid morphology, growth rate, and metabolic activity (Tao et al., 2019; Li X. et al., 2020). While preserving the genetic characteristics of the parent tumor, the organoid chip can provide a stable nutrient concentration or oxygen for PDOs and increase cell activity and homogeneity (Li X. et al., 2020). For example, Pinho et al. (Pinho et al., 2021) prepared a low-cost, rapid prototyping microfluidic chip using a widely used micro-milling machining process. The manipulation of fluid flow in the microchannel by linking a syringe pump is a key aspect of the experimental setup utilized. This enables the generation of a precise and measurable gradient of cell density, ultimately leading to the production of organoids with a more uniform size and increased proliferation activity when subjected to continuous flow conditions and optimal cell density. A microfluidic device designed by Dhiman et al. (Dhiman et al., 2020) included four chip-design copies. Each chip unit was divided into six hexagonal columns to form three side-by-side channels. In the central channel, A549 cells were wrapped in the hydrogel, and 3D multicellular tumor spheres of uniform size (35–45 µm) and good shape were obtained within 5 days.

To better use the organoid model for preclinical drug screening, researchers have carefully observed the effects of centrifugation, freezing, and other processes on the stability of PDOs and found that these routine steps may cause cell differences and reduce their sensitivity to drugs (Yu et al., 2019; Liu et al., 2021). Recently, Liu et al. (2021)) designed a superhydrophobic microporous array of organoids-on-a-chip for *in situ* cryopreservation and subculture of PDOs (Figure 3A). This design eliminated the PDOs damage caused by slow freezing, omitted the centrifugation and resuspension steps, minimized the consumption of PDOs, preserved the vitality of organoids, and proved that the sensitivity of anticancer drugs has not changed, and has repeatability and accuracy. Such advancements in organoid technology are crucial for preclinical drug screening.

#### 3.2.2 Highly simulated TME

The TME, consists of nonneoplastic host elements and neoplastic cells, which work together to promote the development, growth, and spread of cancerous tumors. The complex tumor microenvironment is composed of various components, such as mesenchymal cells (pericytes and fibroblasts), the existing or infiltrating vascular structure (endothelium), and a network of immune cells (Yuki et al., 2020) (T and B cells). The TME is an important factor that directly affects the accuracy of drug response prediction and has become the focus of drug research and development, such as chemotherapy and immunotherapy for lung cancer. Researchers are committed to exploring organoid co-culture, which is a new approach to studying tumor immunobiology (Yuki et al., 2020). Recent reports point to (Dijkstra et al., 2018) use peripheral blood lymphocytes co-cultured with autologous lung cancer PDOs and immunotherapy to evaluate the sensitivity of tumor-like organoids to T cell lethality, the success rate of this study was only about 33%–50%, it is hard to evaluate the feasibility of this method, the co-culture technology of lung cancer PDOs should be developed to reconstruct TME *in vitro* (Bar-Ephraim et al., 2020).

Compared with the traditional PDOs culture systems, microfluidic technology can accurately control the physical and chemical parameters of the equipment on the nanometer scale, reshape the physical and chemical properties of the TME, and simulate the complex interactions between cells in the TME (Park et al., 2019; Duzagac et al., 2021). Therefore, the co-culture of tumor and immune cells from patients on organoids-on-a-chip can overcome the limitations of traditional PDOs function tests. Kitajima et al. (2019) successfully added a foreign immune cell subgroup to the culture medium, such as T cells (Jurkat cells), to explore the direct relationship between tumor cells and Jurkat T cell recruitment defects, which has important guiding value for revealing the potential mechanisms of disease and developing new drugs. Additionally, the microfluidic device developed by Poletti et al. (2021) can combine PDOs with complex bacterial communities specifically separated from the host. This culture mode retains the parent TME components, regularly delivers fresh nutrients, removes dead cells that fall off from the culture cavity through perfusion, prolongs the life of organoid organs, and can achieve the model construction of “host cell-microorganism” interactions to a certain extent, promoting the development of microbiome-based therapy. In addition, the unique physical and chemical environment of TME *in vivo*, such as hypoxia and low nutrition formed in the chip, leads to a subsequent change in the gene expression profile of tumor cells, which is consistent with the altered gene expression observed in patients, indicating that the microfluidic chip highly reproduces the structure and function of TME *in vivo*, such as Kim (Kim et al., 2022)et al. construct brain tumor microenvironment (b TME) to clarify the potential subtle interaction between brain metastatic NSCLC cells and b TME (Figure 3B).

#### 3.2.3 Vascularization

Vascularization of organoids is an innovative technology, which are miniature three-dimensional models of organoids, with microfluidic chips that mimic the structure and function of blood vessels, and highly reproduce the dynamic process of the tumor angiogenesis microenvironment and interaction between cells *in vitro* (Zhao et al., 2021), which is conducive to studying relevant molecular mechanisms and drug analysis (Nashimoto et al., 2017; Homan et al., 2019).

Blood vessels play a crucial role in connecting organoids and facilitating material exchange between them, while also maintaining the metabolic and environmental stability of tissues through micro-vascular networks. The incorporation of vascularization into organoids-on-a-chip technology provides an excellent platform for studying the physiological barrier function of blood vessels, the effects of blood flow shear force on vascular pathology, and substance transport between tissues, as well as the potential for vascular regeneration. For example, In 2014, Takebe et al. (Takebe et al., 2014) utilized a similar method to produce liver organoids that were vascularized. They grew hepatic endoderm cells derived from human iPSCs, as well as HUVECs and hMSCs, which self-organized to form liver buds (iPSCs-LBs). These were then transplanted under the cranial window in mice, and the results showed that human blood vessels connected to the host vessels, creating unobstructed conduits that were able to transport oxygen and (Takebe et al., 2013). This study demonstrates that the transplantation of iPSCs-LBs cultured *in vitro* into a host can produce vascularized liver organoids that possess a functional tissue architecture. Overall, this technology offers a valuable tool for investigating the behavior and function of blood vessels in various contexts, and drug screening for compounds, which inhibit tumor angiogenesis (Skardal et al., 2017).

#### 3.2.4 Dynamic monitoring of drug reactions

In terms of drug efficacy assessment, traditional methods usually involve staining PDOs and discriminating cell viability and status using fluorescence microscopy (Wu et al., 2018). This method is not only complicated and affects subsequent experiments, but it is also difficult to achieve dynamic real-time monitoring of organoids.

Microfluidic technology originated from the manufacture of integrated circuit chips, from which the introduction of sensors can dynamically and continuously monitor the response of similar organoids to drugs without intervention and destruction (Sontheimer-Phelps et al., 2019). For example, combining a microfluidic chip and a high-resolution imaging system can realize dynamic detection of the activity and number of organoids. Schuster et al. (2020) coupled a microfluidic chip with an electrical sensor to establish an integrated chip for real-time efficacy evaluation. By detecting the overall impedance of the gel and organoid, the chip could quickly and accurately reflect the impedance changes of tumors after the action of chemotherapy drugs and then judge the response of tumor cells to different drugs. Compared to conventional drug analysis, the chip could quickly identify drug-sensitive and drug-resistant cells within 12 h. Cells were inoculated onto the surface of the sensor through multi-channel micro-nano sensors to monitor the real-time state of the cells.

Deep learning and cross-scale fusion of microfluidic chips capture the inherent characteristics of data more efficiently and accurately to achieve cell recognition, location, tracking, and image segmentation, which are powerful monitoring tools for the further study cell-cell interactions in the TME and drug treatment response (Mencattini et al., 2020). For example, Hashemzadeh et al. (2021) combined microfluidic technology with a deep learning algorithm and classified 3D lung cancer cells (P-C9, A-427, A-549, and others) cultured in the microfluidic platform for the first time under the condition of simple single-cell operation, simultaneous multiple analysis, and only a small number of samples (microliter range). The success rate was more than 90%. This model is expected to help pathologists detect cancer cells in conventional pathological practices, thereby reducing the burden of pathologists.

Monitoring cell proliferation and apoptosis by imaging is not sufficiently comprehensive for personalized drug screening. This is the most important application of organoid microarrays. For example, for drugs that act on tumor cells or the microenvironment, we need to recognize different subtypes of tumor cells or look for new biomarkers to screen people who are sensitive to a specific treatment to use drugs accurately for that population. Therefore, the content of various ions and protein macromolecular markers in the organoid culture microenvironment is an important indicator in drug screening. Organoids based on microfluidic technology may achieve multiparameter dynamic detection, which can more comprehensively and accurately evaluate the efficacy or toxicity of drugs. For example, by connecting a light-addressable potential sensor (LAPS) or using a fluorescent probe, it is possible to continuously observe various parameters, such as protein macromolecules, mRNA, pH, electrochemical changes, and others with high sensitivity, without disturbing the organoid culture process (Zhang et al., 2017). The convenient real-time and accurate reliability of LAPS has accelerated medical scientific research.

#### 3.2.5 Achieve high-throughput drug screening

One of the major obstacles for generating organoids is the formidable task of procuring an adequate number of viable cells from patient samples. Moreover, traditional organoid culture systems are intricate to operate manually, and lack the ability to simulate the most frequent periodic dosing schedules used in clinical practice. Consequently, the challenge remains to achieve automation and high-throughput drug screening (Schuster et al., 2020).

Compared with the traditional perforated plate, the nanoscale cultivation capacity and multi-channel parallel design on the chip greatly reduce the number of requirements for PDOs cultivation, can react quickly in the microchannel or microcavity, and has low reagent consumption and simple manual operation, which greatly reduces the cost (Schuster et al., 2020). Au et al. (Au et al., 2014) developed an organoid drug screening platform based on microfluidic technology. This platform can control the movement of single-cell gel clusters, and each liver cancer microsphere with good homogeneity can be regarded as an independent liver cancer culture unit, which greatly increases the parallel processing ability of the analysis unit in the chip and improves the reaction speed of the drugs in the organoids-on-a- chip. Liu et al. (2021), Kim et al. (2022), and Kim S. K. et al. (2021) used resected lung cancer tissues to analyze the whole gene sequence of PDOs on the successfully constructed organoid microarray. The results show that the detection of lung cancer PDOs and organoids can not only reduce the amount of organoid culture, but also improve the experimental flux and overcome the key problem of small clinical sample size. Compared with traditional organoid culture methods that require a large number of manual drug deliveries, the use of mechanical and automated organoid chips can reduce time-consuming and laborious liquid transfer steps, avoid human errors as much as possible, and reduce the failure rate (Schuster et al., 2020). The device can also easily combine various drugs at different concentrations and realize a series of functions, such as large-scale, multi-drug processing, and identification of potential drug combinations. For example, Zhai et al. (2021) invented a droplet injection device for delivering drug dosage. Hundreds of cell microchannels were designed on the chip. Tumor cells of different densities were inoculated into microchannels. Through the automatic distribution of drugs on the chip, drug concentrations ranging from three to four orders of magnitude were generated. According to the drug screening requirements, different types and concentrations of chemotherapy drugs can be injected into the chip, greatly simplifying the early and complex drug screening process and improving the efficiency of drug research and development.

#### 3.2.6 Multi-organoids interactions

The complexity of the human body arises from intricate interactions between components at different organizational levels. While organoids can approximate native cell types and their communication, modeling higher-level biological interactions is challenging. One potential solution to this problem is organoid-on-a-chip technology.

By co-culturing different organoid types in a compartmentalized microenvironment, it is possible to create a multi-organoid system that mimics complex physiological processes and systemic responses to external stimuli (Park et al., 2019). Recently, a multi-organ chip was developed that featured matured human heart, liver, bone, and skin tissue niches linked by vascular flow (Ronaldson-Bouchard et al., 2022). The chip recapitulated interdependent organ functions in the presence of endothelial barriers and was successfully utilized for drug testing. Similarly, Yin et al. (2021) presents a novel multi-organoid-on-a-chip platform composed of hiPSC-derived organoids for the safety evaluation of an antidepressant drug, clomipramine. The bioengineered system is fabricated with four layers, enabling the 3D co-culture of liver and heart organoids in separate chambers, following self-organization of hiPSC-derived organoids. The upper multi-well chamber is dedicated to liver organoid culture to assess the metabolism of clomipramine. This is the first time that such a system has been developed for the safety assessment of antidepressants using organoids-on-chips technology. This novel platform enables the reflection of drug metabolism and responses at the multi-organ level *in vitro*, providing a means for drug effectiveness and toxicity assessment.

**FIGURE 2:**
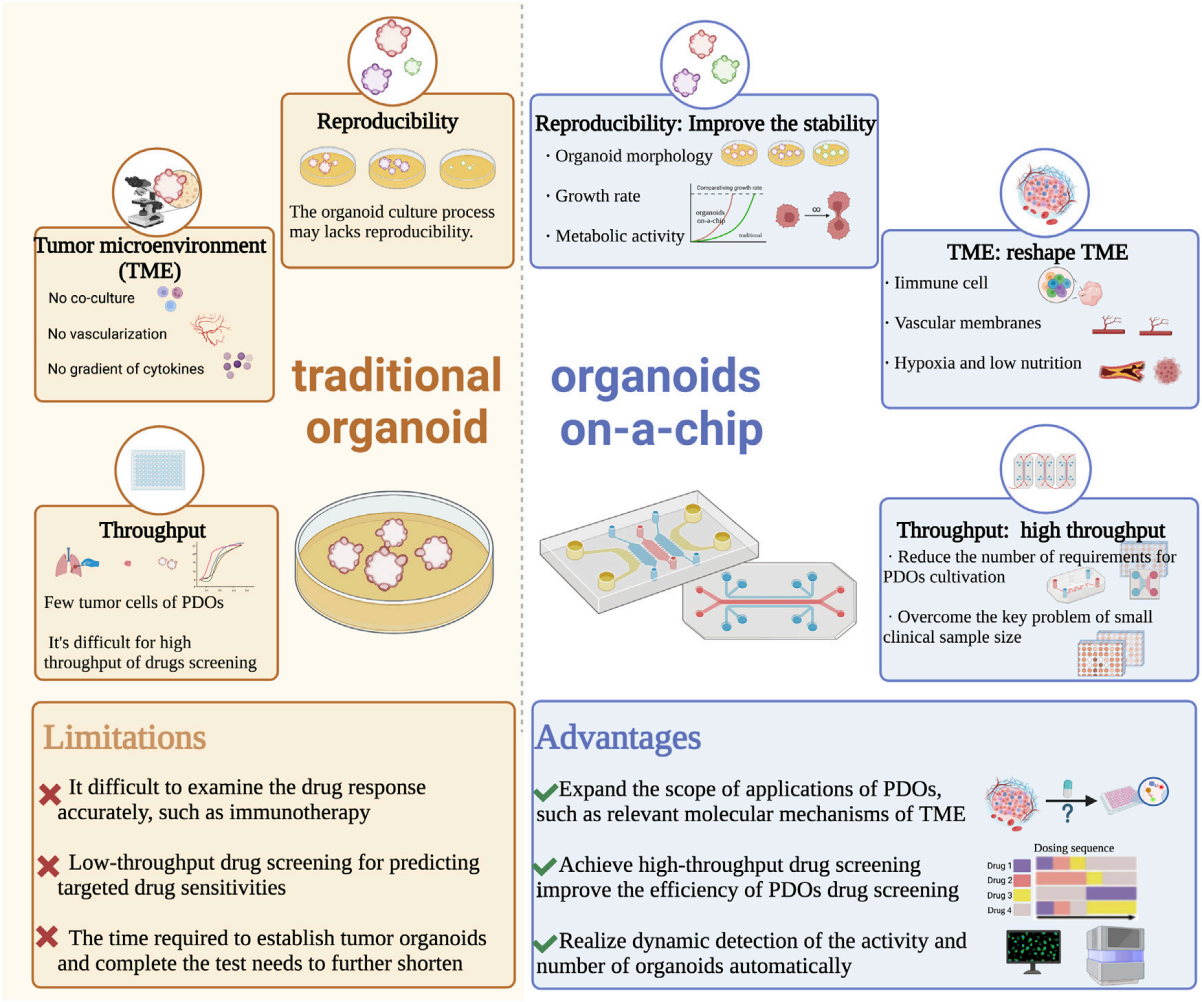
Comparison of traditional organoid and organoids on-a-chip in the developments and applications of PDOs. Abbreviations: PDOs, patient-derived organoids; TME, tumor microenvironment.

In a summary, the unique advantages of microfluidics technology in controlling and manipulating small amounts of fluids and cells have improved the tumor microenvironment in organoid cultures and enabled subsequent high-throughput, automated drug sensitivity experiments. This creates new opportunities for personalized diagnosis and treatment of lung cancer (Figure 2).

## 4 Research progress of organoids-on-a-chip in precise treatment of lung cancer

In recent years, advances have been made in the organoids-on-a-chip and microphysiological systems for drug screening, disease modeling and personalized medicine. We searched all literature related to the development, diagnosis, and treatment of lung cancer using organoids-on-a-chip. Considering that the concept of organoids-on-a-chip has been proposed for less than 5 years, we also expanded the scope of the search to include studies related to microfluidics chips. Organoids-on-a-chip is developed based on microfluidics chips. Although there are some differences in the design of chip channel structures (microfluidics chips have larger chambers that can accommodate multiple organoids with no organoid size control), some scholars have used such devices to cultivate 3D organoids by hydrogels composed of Matrigel or similar basement membrane extracts to microchannels (Lancaster et al., 2017; Bircsak et al., 2021; Yin et al., 2021). Undoubtedly, microfluidics chips and organoids-on-a-chip represent two different yet complementary approaches to achieving the same goal of replicating the complexity of human organs *in vitro*, which has valuable implications for the precision treatment of lung cancer based on organoids-on-a-chip. For clarity, we limit the use of the term “microfluidics chips” to describe the microfluidics chip without necessarily using 3D organoids, while retaining the use of the term “organoids-on-a-chips” to represent organoid-based microfluidic technologies.

### 4.1 The high reliability and efficiency of personalized drug screening

#### 4.1.1 Precise control of organoid culture for its stability

**FIGURE 3:**
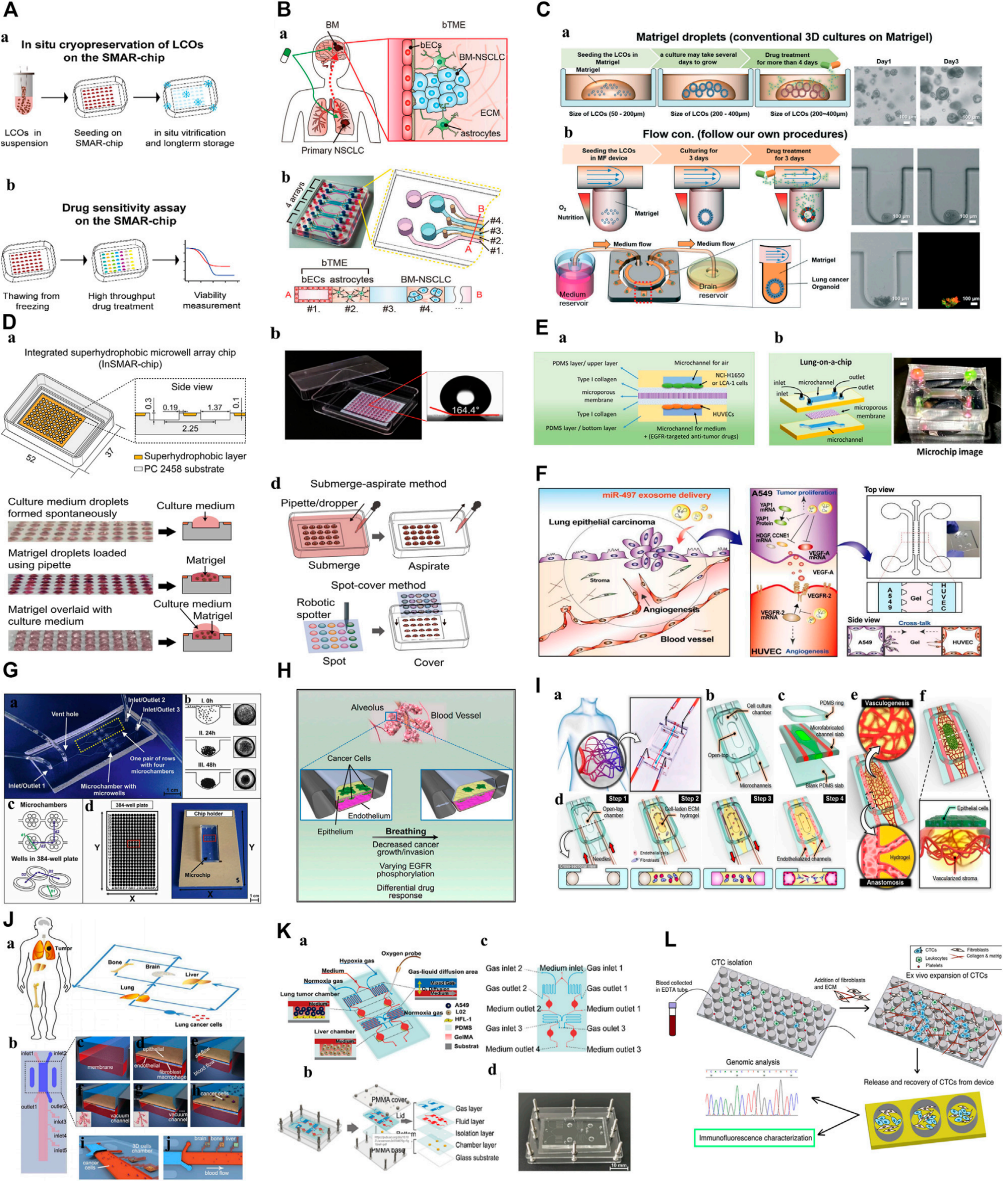
Microfluidic Organoids-on-a-Chip platform. **(A)**: Diagram of the *in situ* freeze–thaw cycle and the subsequent drug sensitivity test on the SMAR-chip (Liu et al., 2021). “Adapted with permission from (Liu et al., 2021). Copyright 2021by the authors.” **(B)**: Schematic illustration of brain metastatic niche of NSCLC cells and microfluidic device for recapitulating the niche. a. The brain metastatic niche involving bTME with bECs and astrocytes. b. Representative photography and drawing of the microfluidic device with seven channels (Kim et al., 2022). “Adapted with permission from (Kim et al., 2022). Copyright 2022 The Authors.” **(C)**: Schematic diagram of the microfluidic device for lung cancer organoids. a. Patient-derived LCOs were cultured and expanded in Matrigel droplets in 24-well plates. b. The YFR passive microflow device allowed for a uniform distribution of the organoids (Jung et al., 2019). “Adapted with permission from (Jung et al., 2019). Copyright The Royal Society of Chemistry 2019.” **(D)**: Characterization of the InSMAR-chip. a. Schematics of the InSMAR-chip and the cross-section view of the chip. b. Photograph of an InSMAR-chip with a droplet array in the microwells. c. Photographs of the droplets in the microwells. d. Schematics of the reagent delivery methods on the InSMAR-chip (Hu et al., 2021). “Adapted with permission from (Hu et al., 2021). Copyright The Author(s) 2021.” **(E)**: a. A schematic diagram of the experimental principle. b. A schematic diagram and image of the lung-on-a-chip (Tan et al., 2022). “Adapted with permission from (Tan et al., 2022). Copyright 2022 by the authors.” **(F)**: Schematic illustration showing the overall experimental strategy for delivery of miR-497-loaded exosome in an *in vitro* NSCLC model (Jeong et al., 2020). “Adapted with permission from (Jeong et al., 2020). Copyright The Royal Society of Chemistry 2020.” **(G)**: 3D lung spheroid cultures for evaluation of PDT procedures in microfluidic Lab-on-a-Chip system. a. The fabricated microchip. b. The scheme of cell aggregation and spheroid formation in the microwell. c. The scheme of microchamber arrangement. d. The chip holder with the microchip placed and comparison with the 384-well plate (Zuchowska et al., 2017). “Adapted with permission from (Zuchowska et al., 2017). Copyright 2017 Elsevier B.V.” **(H)**: This model reproduces the organ microenvironment-specific cancer growth, tumor dormancy and response to TKI treatment observed in human patients (Hassell et al., 2017). “Adapted with permission from (Hassell et al., 2017). Copyright 2017 The Authors.” **(I)**: A microengineered *in vitro* 3D culture platform to produce self-assembled and perfusable microvascular beds (Paek et al., 2019). “Adapted with permission from (Paek et al., 2019). Copyright 2019, American Chemical Society.” **(J)**: Schematic illustration of the multi-organ microfluidic chip, which includes an upstream “lung organ” and three downstream “distant organs” (Xu et al., 2016). “Adapted with permission from (Xu et al., 2016). Copyright 2016, American Chemical Society.” **(K)**: 3D-CMOM platform. a. Schematic diagram displays the functional description of each area of the platform. b. A diagram of the multilayer structure that comprises the platform. c. The details of the function of each hole in the platform. d. Image of the microfluidic platform (Zheng et al., 2021). “Adapted with permission from (Zheng et al., 2021). Copyright 2021, American Chemical Society.” **(L)**: Diagram of the expansion of CTCs from early stage lung cancer patients using a microfluidic co-culture model (Zhang et al., 2014). “Adapted with permission from (Zheng et al., 2021). Copyright 2014 Zhang et al.” Abbreviations: NSCLC, non-small cell lung cancer; bTME, brain tumor microenvironment; bECs, brain endothelial cells; BM, brain metastases; LCOs, lung cancer organoids; PDMS, prepared polydimethylsiloxane; EDTA tube: Anticoagulation tube; CTC, circulating tumor cells; 3D-CMOM, three-dimensional-culture multiorgan microfluidic; PMMA, polymethylmethacrylate; YFR, yarn flow resistor; InSMAR-chip, integrated superhydrophobic microwell array chip; PDT, photodynamic therapy; TKI, tyrosine kinase inhibitor.

Organoids-on-a-chips enables precise and dynamic handling of fluids and tissues at the biological length scales of organoids, affecting physiological conditions such as organoid morphology, growth rate, and metabolic activity for stability, with the potential to contribute to reducing the high level of drug discovery failure. This reliable modling improves the credibility of organoid related experimental results. A good example can be found in the one-stop microfluidic system was developed by Jung et al. (2019), which delivers nutrition and oxygen to the lung cancer PDOs through the continuous flow of drug-containing culture medium to maintain the shape and function of organoids (Figure 3C), increasing stability and viability of organoids when compared to traditional cell culture methods. And what’s interesting is that then researches conducted the dynamic drug sensitivity tests, after the induction of cisplatin and etoposide, the cells in the peripheral area of the lung cancer PDOs die, while the cells in the core area can survive for 7 h. This proves that the core area of PDOs contains chemoresistant cells, indicating that the system can better predict the drug response of lung cancer and select the best treatment plan. This accuracy and consistency of mechanical automation offer a means to reduce the variability resulting from irregular manual manipulation in laborious laboratory processes (Au et al., 2014).

#### 4.1.2 High-throughput drug screening of lung cancer organoids

In lung cancer, increasing the density at which organoids can be cultured and for drug test is important factors affecting the efficacy of drug screening, organoids-on-a-chips provides the opportunity. The ability to select, manipulate, and screen PDOs in a high-throughput manner has the potential to be advantageous for achieving high drug screening efficacy. For example, Hu et al. (2021) developed a superhydrophobic micropore array chip based on lung cancer PDOs, which can generate hundreds of organoids in a short time and conduct drug sensitivity tests of high-flux chemotherapy drugs (Figure 3D). It is worth noting that this is the first time that lung cancer PDOs has been introduced into the chip for high-throughput screening with different concentrations within 1 week. This shortens the time of organoid culture and drug testing, solves a major problem of accurate diagnosis and treatment, and greatly accelerates the clinical transformation efficiency of PDOs applications. On this basis, organoids-on-a-chip can also achieve drug combinations for high-throughput manipulation. Tan et al. (2022) prepared polydimethylsiloxane (PDMS) lung cancer organoids-on-a-chips using advanced 3D printing technology, which can screen single or combined drugs in order. 3D bioprinting technology, as a manufacturing technique, offers several advantages including unlimited design space, the freedom to create complex geometries, and a reduction in the need for manual manipulation during tedious laboratory procedures such as organoid culture and high-throughput drug delivery (Monteiro et al., 2022; Neufeld et al., 2022). This is significant for the process of testing large numbers of drugs on multiple organoids simultaneously. (Figure 3E). In a summary, this platform provides a robust, novel model which can be used for high-throughput drug screening, enhancing clinical and translational applications.

#### 4.1.3 Integration of biosensing for automatic analysis

Another benefit of using microengineered systems for personalized drug screening of lung cancer PDOs is that efforts to address efficiency can be greatly facilitated by incorporating biosensing elements into culture platforms, which enables continuous screening of organoids (Choo et al., 2021), integrating and generating molecular interactions of specific tumor-associated proteins in lung cancer and perform large-scale validation of predicted proteins in PDO

Tan et al. (2022) developed a robust and streamlined automated microfluidic chip that allows high-throughput culture, stimulation, assaying, and harvesting of organoids under dynamic conditions. Which can screen single or combined drugs (EGFR-targeted drugs, including gefitinib, afatinib, and osimertinib on NCI-H650 cells and primary lung cancer cell, then monitor drug response indicators in real time. This system featured a fluorescence photography platform capable of showing the change in fluorescence intensity more intuitively with high sensitivity and wide dynamic ranges. Recently, some researchers (Wiedenmann et al., 2021) have been able to carry out automatic structural digestion for organoids, conduct multi-level heterogeneity analysis and interpretation at the single-cell level, achieve single-cell distribution and database building on the chip, and then achieve drug response differential clustering and single-cell type classification based on sequencing data analysis, proving that PDOs can replace human lung cancer for *in vitro* research, and is of great significance for promoting individualized treatment of lung cancer.

### 4.2 Applications in innovative drugs and therapies for lung cancer

Although many advances have been made in the treatment of lung cancer, it remains a major health problem worldwide. In addition to prevention and early diagnosis, more innovative therapies are needed for cancer treatment. Microfluidic chip will partially address the problem mentioned, facilitating translation to industry. For example, a 3D microfluidic device was co-cultured with human umbilical vein endothelial cells (HUVEC) designed by Jeong et al. (2020) (Figure 3F). It explored the antitumor and antiangiogenic effects of miR-497 in the TME of NSCLC. The results showed that, compared with the control group, tubular formation of endothelial cells and tumor migration were significantly reduced. This shows that exosome-mediated miRNA therapy combined with microfluidic technology can become a predictive and cost-effective clinical transformation research tool for targeting tumor growth and angiogenesis, and can provide new clinical treatment options for lung cancer patients. Dhiman et al. (2020) performed similar experiments, providing a new treatment strategy for lung cancer cells (a Novel Trp-Rich Peptide) without affecting healthy cells, suggesting that the 3D microfluidic chip used may help to develop models of several other cancer types to test a variety of new anticancer drugs.

Interestingly, Sankar et al. (2021) used surgically resected tissues from lung cancer patients for organoid culture, screened the efficacy of three chemotherapeutic drugs according to different combinations (three monotherapy, three paired combinations, and three drug treatments), and used a novel U-shaped hole 3D microfluidic chip. Under the condition of preserving some important characteristics of tumor tissue *in vivo* (3D cell structure and genetic information, dynamic fluid flow, etc.), it has been determined that low-dose rhythm chemotherapy may reduce the side effects of drugs and provide better therapeutic effects for cancer patients.

Zuchowska et al. (2017) conducted a study of 5-aminolevulinic acid (ALA-PDT) on a 3D lung cancer model in a microfluidic system for the first time (Figure 3G). This method can be used to determine safe drug concentrations and PDT parameters. In the future, it can be used as a suitable and effective preclinical model to guide clinical drug use, which is important for conducting *in vitro* drug testing and realizing new treatments.

### 4.3 Explore the complex interaction and mechanism in lung cancer for understanding cancer progression

#### 4.3.1 Lung cancer TME

To simulate the local microenvironment of tumor drug delivery, the researchers used lung cancer cells and fibroblasts to inoculate the corresponding microchannels according to the spatial distribution characteristics *in vivo* and constructed an organoid chip co-culture system to capture the interaction between the tumor cell population and matrix components. This study found that fibroblasts gradually formed clusters and promoted the proliferation of tumor cells. On this basis, it was confirmed that the co-culture of tumor cells and fibroblasts was less sensitive to gefitinib and cisplatin (Xu et al., 2013). Similarly, Kitajima et al. (2019), Hassell et al. (2017) conducted similar studies (Figure 3H). For example, by adding Jurkat cells to the medium channel, the interaction and crosstalk between Jurkat cells and tumor cells in the process of infiltration into the tumor sphere can be further studied, revealing that the mechanism of PD-1 inhibitor treatment for primary drug resistance is closely related to the depletion of T cells around the TME. The results of this study show that the chip not only successfully simulates the immunity of TME *in vivo* but also helps to identify the key regulatory factors of tumor growth and invasion in TME, so as to conduct the corresponding drug research. Similarly, Ying et al. (2015) studied the effects of CAF and paclitaxel on Met/PI3K/AKT activation and GRP78 expression using a double-layer 3D perfusion cell culture microfluidic device integrated with a concentration gradient generator, indicating that the device is an ideal platform for signal research and drug screening.

#### 4.3.2 Lung cancer vascularization and metastasis

Anti-tumor metastasis is also a challenge and a research hotspot for targeting the lung cancer cancer progression and resistance, and microfluidic technology has become a new way to build lung cancer metastasis models. Its main research topics include single-cell analysis, endothelial cell migration, and neovascularization (Ruzycka et al., 2019). A constant flow rate microfluidic device was designed by Lee et al. (2019). They co-cultured lung cancer cells, vascular endothelial cells, and type I collagen matrix to simulate the TME *in vivo*. It was found that the continuous flow of cell culture media greatly affected the proliferation of cancer cells in micropores and the integrity and sustainability of the endothelial cell layer in the microfluidic channel, and further explored the response of the 3D culture system to matrix metalloproteinase (MMPs) inhibitors. We found that MMPs inhibitors prevented the separation and contraction of the surrounding collagen matrix, improved the stability of the collagen matrix, and achieved the goal of preventing tumor metastasis. In addition, Paek et al. (2019) recreated the vascularization of a 3D culture of human lung adenocarcinoma (Figure 3I). The chip device inoculated lung adenocarcinoma cells and endothelial cells into a cell culture chamber with a top opening and two parallel microchannels used to control vascular perfusion, respectively, to study the structurally variable microvessels in the microenvironment and evaluate the intravascular delivery characteristics of paclitaxel. With the help of the vascular system, the number of apoptotic cells in the organoid center increased, and the average vessel diameter reduced in length and density.

Anguiano et al. (2017) studied the migration phenotype of lung cancer cells in different TME matrices using microfluidic chips and integrated images. The results showed that cell migration may be related to matrix stiffness and pore size, and the change in migration speed is related to the change in migration phenotype. It can be seen that the microfluidic system is of great significance for exploring the migration of lung cancer and studying the treatment under the biological background.

Xu et al. (2016) designed a microfluidic chip that can simulate the invasion microenvironment of lung cancer and the interaction between cells and the cells/matrix (Figure 3J). The lung cancer cells cultured in this device form a three-dimensional sphere and display epithelial-mesenchymal transformation (the expression changes of E-cadherin, N-cadherin, Snail1, and Snail2), which is further used to evaluate the possibility of invasion of distant organoids (brain, bone, and liver). On this basis, the team identified the potential mechanism of acquired drug resistance in brain metastases (BM) and provided a new strategy to overcome drug resistance in lung cancer BM(Xu et al., 2020).

Zheng et al. (2021) designed a 3D culture multiorgan microfluidic device with precise control of dissolved oxygen concentration, successfully constructed a lung cancer metastasis model under hypoxia, studied the mechanism of promoting cancer metastasis under hypoxia and evaluated HIF-1α Cancer treatment effect of inhibitors (tirapazamine, SYP-5, and IDF-11774) (Figure 3K). Jin et al. (2019) conducted a similar study and found that only under hypoxia, Netrin-1 mediated epithelial-mesenchymal transformation (EMT) of a lung cancer 3D culture model *in vitro* was associated with the phosphoinositol 3 kinase/AKT pathway. Therefore, the dynamic process of tumor-mesenchymal cell interaction, angiogenesis, and tumor metastasis in the TME can be reproduced *in vitro* using organoid chips, which is conducive to the study of relevant molecular mechanisms and drug analysis, and further promotes the precise treatment of lung cancer.

### 4.4 Use of circulating tumor cells for microfluidic 3D co-culture

Circulating tumor cells (CTCs) are tumor cells that shed from solid tumors and invade peripheral blood circulation. They are also used as biomarkers for liquid biopsy. Owing to their convenient sampling and small trauma, they play an important role in early diagnosis, prediction of tumor development, and guidance of individual chemotherapy (Paek et al., 2019; Li et al., 2022). However, because the concentration of CTCs in peripheral blood is lower than that of other blood cells, and the ratio of the two is approximately 1:109 (Jansson et al., 2016), it is difficult to achieve effective *in vitro* amplification of very few target CTCs using the commonly used liquid histological examination methods (such as density gradient centrifugation, filtration, and immunomagnetic separation). This results in limited clinical application (Cho et al., 2018; Farshchi and Hasanzadeh, 2021). In recent years, the separation technology of CTCs based on microfluidic chips has been widely studied because it significantly improves the separation flux of CTCs and increases their detection sensitivity and specificity (Praharaj et al., 2018).

Zhang et al. (2014) successfully constructed an 3D microfluidic chip model co-cultured with fibroblasts using the whole blood of 19 patients with early lung cancer (the success rate was 73%). It can complete the expansion of CTCs as tumor spheres *in vitro* within 14 days, which is used for the analysis of genes, proteins, and functions at all levels, and has important clinical significance for tumor precision medicine (Figure 3L). This method solves the problem of sorting, amplification, and 3D culture of CTCs in a one-stop manner. CTCs cultured and expansion in microfluidic chips can also be used for animal model construction, drug resistance, and drug sensitivity analysis. However, we need to clarify that tumor spheres and organoids belong to the same 3D culture system, the biggest difference between them is that tumor spheres grow in a scaffold-free system (e.g. 3D gel), while organoids mainly grow in a scaffold-based system (e.g. matrigel). The aim of this discussion is to assess the utility of organoids-on-a-chip and microfluidic chips in the precise management of lung cancer. Distinct growth conditions required for tumor spheres and organoids do not compromise the advantages of microfluidic chips in enabling accurate manipulation and realistic simulation. Therefore, this research which has integrated CTCs systems with organoids is valuable for early diagnosis, prediction, and guidance of individual therapy.

In a summary, with the help of the unique advantages of microfluidic technology in the control and treatment of trace liquids and cells, it not only improves the TME of organoid culture, but also realizes follow-up high-throughput and automatic drug sensitivity testing. This creates a new opportunity for the diagnosis and treatment of lung cancer (Table 2).

**TABLE 2:** Research progress of 3D microfluidic chip and organoids-on-a-chip in accurate treatment of lung cancer.

Sample source	Characteristics of microfluidic chip (structure, material and process)	Application and discovery	References
Surgical resection of tissue	Three culture chambers and four medium channels form a microfluidic unit, totaling four units	1. To clarify the potential interaction between brain metastatic NSCLC and bTME	Kim et al. (2022)
		2. Sensitivity test of clinically targeted BM drugs (alfatinib, PKI587, seretinib and trimetinib, etc.)	
Surgical resection of tissue	Preparation of PDMS lung organ chip by 3D printing technology	Clinical EGFR targeted drugs test (gefitinib, alfatinib and oxitinib)	Tan et al. (2022)
Surgical resection of tissue/biopsy tissue	Superhydrophobic microporous array containing TiO2 nanoparticles (SMAR-chip)	Sensitivity test of clinical targeted/chemotherapy drugs (tyrosine kinase inhibitor, paclitaxel, cisplatin, etc.)	Hu et al. (2021)
Surgical resection of tissue	Superhydrophobic microporous array containing TiO2 nanoparticles (SMAR-chip), Vitrification freezing kit	1. *In situ* freezing and thawing of PDOs	Liu et al. (2021)
		2. Chemotherapy drug sensitivity test (gemcitabine, pemetrexed or doxorubicin, etc.)	
Surgical resection of tissue	Porous structure channel, polycarbonate optimized filling	Sensitivity test of clinical chemotherapy drugs (gemcitabine and cisplatin)	Kim et al. (2021a)
Biopsy tissue, pleural effusion	One-stop integrated microfluidic	1. Detection of organoid status (uniform growth)	Jung et al. (2019)
		2. Sensitivity test of clinical chemotherapy drugs (cisplatin and etoposide)	
Blood sample	Microfluidic chip based on 3D co-culture	1. Select and expand circulating tumor cells from blood of patients with early lung cancer	Zhang et al. (2014)
		2. It can carry out gene and protein functional analysis and has the potential to discover new biomarkers	
Lung adenocarcinoma cells (H1975)	Culture of lung cancer cells and vascular endothelial cells with porous micro-ECM coated plate	1. Simulate the development and invasion of EGRF mutant lung cancer cells	Hassell et al. (2017)
		2. Sensitivity test of clinical targeted drugs (tyrosine kinase inhibitor)	
Lung adenocarcinoma cells (A549)	3D cell culture chamber with top opening and 2 parallel microchannels for controlling vascular perfusion	1. Self-assembled and perfusion microvascular network	Paek et al. (2019)
		2. Vascular toxicity test of clinical chemotherapy drugs	
Lung adenocarcinoma cells (A549)	Multi-organ microfluidic chip (upstream chamber simulates lung; downstream chamber simulates brain, bone and liver)	Construction of lung cancer metastasis model and validation *in vivo*	Xu et al. (2016)
Surgical resection of tissue	A novel microfluidic chip with a U-shaped hole based on PDMS	Screening of low-dose metronomic chemotherapy drugs (paclitaxel, vinorelbine and etoposide)	Sankar et al. (2021)
Lung adenocarcinoma cells (A549)	The A549/HUVECs cultured on one side of the channel and VEGF-A added to the opposite side of channel across the gel channel	To investigate the anti-tumor and anti-vascular effects of NSCLC in 3D microfluidic device using microRNA-497 therapy	Jeong et al. (2020)
Lung adenocarcinoma cells (A549)	the multilayer structure (the gas, fluid, and isolation layers) of lung cancer metastasis model under hypoxia that comprises the platform	1. Study the mechanism of promoting cancer metastasis under hypoxia	Zheng et al. (2021)
		2. Evaluated HIF-1 α Cancer treatment effect of inhibitors (tirazamine, SYP-5 and IDF-11774)	
Lung adenocarcinoma cells (A549)	Cancer cells were seeded in the microfluidic chip with passive flow pump	Explore the dose response of matrix metalloproteinases MMPs inhibitors	Lee et al. (2019)
Lung adenocarcinoma cells (A549) and (PC9)	a gel micro-channel running between two micro-channels, each with two rows of micro-gaps	Explore the mechanism of hypoxia	Jin et al. (2019)
Lung adenocarcinoma cells (A549)	The microfluidic chip with concentration gradient generator and downstream cell chambers (the upper panel) and the fabricated chip with pumping machine (the lower panel)	An ideal platform for signaling research and drug screening	Ying et al. (2015)
Lung adenocarcinoma cells (A549)	Four rows of microchambers with a network of microchannels. Each microchamber contains seven microwells with a spherical shape	It is the first study of photo-driving therapy (ALA-PDT) of 3D lung cancer model in microfluidic system, and the appropriate drug concentration and PDT parameters can be found	Zuchowska et al. (2017)
Lung adenocarcinoma cells (A549)	cell-laden (A549) hydrogel in the central channel (1,000 µm wide) and media in the two side channels (each 1,000 µm wide)	The microfluidic device used may help develop models of several other types of cancer to test new anticancer drugs	Dhiman et al. (2020)
Lung adenocarcinoma (H1299)	Characterization of three-dimensional cancer cell migration in mixed collagen-Matrigel scaffolds using microfluidics	1. To study the migration phenotype of lung cancer cells in the matrix of different TME	Anguiano et al. (2017)
		2. The confocal reflection microscope was integrated and revealed that migration speed changes are linked to migration phenotype changes	
(PC9) cells	Multi-organ microfluidic chip (upstream chamber simulates lung; downstream chamber simulates brain, bone and liver)	Find out the potential mechanism of acquired drug resistance in BM	Xu et al. (2020)

## 5 Prospect of lung cancer PDOs based on organoids-on-a-chip for clinical and translational applications

Today, the application of organoids-on-a-chip has pointed the way for precise treatment of lung cancer to a certain extent. This has turned the dilemma of transforming the basic research results of cancer into effective clinical treatment plans. However, to realize personalized whole-process management of lung cancer patients, a large number of studies by utilizing lung cancer PDOs are still needed to further clarify the molecular characteristics and potential biomarkers in the occurrence and development of lung cancer. Meanwhile, although recent advances in organoids-on-a-chip for drugscreening, there are still lack of standardization to the innovative, reliable, and predictable organoids-on-a-chip models of diseased tissue. Significant work remains to establish stricter quality controls to standardize phenotypic analyses and make organoids-on-a-chip more reproducible.

### 5.1 Explore the mechanism of occurrence and development of lung cancer

The study of early lung cancer, particularly high-risk lung nodules, is of great significance in revealing the causes of lung cancer. However, at present, technical bottlenecks such as the difficulty of animal modeling methods and the long cycle have become crucial limition in the decoding of the mechanism lung nodules/early lung cancer at this stage (Huo et al., 2020). PDOs enabled a better comprehension of the complexity of lung disease initiation and progression, which helps us understand the internal mechanism of tumor development and drug resistance. At the same time, large-scale organoid library construction or biobanking improves the use of drug efficacy tests and the credibility of drug screening results, which is also expected to provide guidance for exploring the pathological types of high-risk lung nodules of patients at different stages of development, this provides a new entry point for construction of a tumorigenesis model, which is of great significance to precise treatment of early lung cancer.

### 5.2 Search for candidate biomarkers

Clear biomarkers are important for the accurate diagnosis and treatment of tumors. Heninger et al. developed an organoids-on-a-chip equipped with biosensors or biological imaging, which can continuously monitor the level of cell metabolism in the culture medium without removing cells from the perfusion culture system, and then judge the different responses of different tumor cells, such as prostate cancer, liver cancer, and breast cancer, to anti-tumor drugs (Choi et al., 2015; Riahi et al., 2016; Heninger et al., 2021) to identify candidate biomarkers of drug sensitivity. For example, the integrated analysis function of the prostate cancer organoids-on-a-chip can rapidly detect fluctuations in prostate cancer-related epigenetic biomarkers (RASSF1, APC, and RARb gene hypermethylation) before and after drug administration. However, studies on lung cancer organoid chips in this field have rarely been reported. Therefore, the universal organoids-on-a-chip will provide us with an integrated platform for real-time evaluation of drug efficacy and detection indicators to quickly and accurately discover potential biomarkers, further demonstrating the application value of the organoid model for lung cancer in finding biomarkers for the population with indications, and promoting the rapid development of accurate treatment of lung cancer.

### 5.3 Optimize the process of lung cancer PDOs drug screening

Testing anti-cancer drugs using PDOs based on organoids-on-a-chip is possible to gain a better drug sensitive, and enable understand the molecular mechanisms of drug efficacy before they are tested in clinical trials. However, generation method and lung cancer PDOs culture (sampling, digestion, biochemical and biophysical microenvironment, and nutrient supply) of organoids-on-a-chip system between groups has become a limitation of research and application. Moreover, drug delivery, reaction detection and result interpretation for providing a reference for patient treatment plans are not mentioned. In addition, the referential steps for the applicability for various types lung cancer of organoids have been released, and thus the standardized protocol and validation methods needs to be promoted. Further optimization of organoids-on-a-chip culture methods is needed to confirm (Lee et al., 2021).

To build a stable and mature lung cancer PDOs drug screening process, it is necessary to optimize the process in combination with the new technology being developed to ensure the vitality of tumor cells and to improve the accuracy and success rate of drug screening (Bengtsson et al., 2021). Based on the general process of tumor organoid drug sensitivity detection combined with organoid chip technology, the drug screening process of lung cancer PDOs can be optimized as follows. 1) Construction of lung cancer PDOs: the tissue with good tumor nature is collected, and the tumor samples are subjected to multi-group analysis for various types of culture methods and medium components, and seeded on the microfluidic chip co-cultured with fibroblasts and vascular endothelial cells for culture with certain biochemical gradients, mechanical forces, nutrients and oxygen. 2) Identification of organoid: optical and metabolic sensors are used to evaluate the growth status of organoid and detect the metabolic level of lung cancer, such as the content of lipid and lactic acid. 3) Drug delivery: flexibly select different drug schemes for testing through micro-channel, such as exploring the sensitivity or toxicity test of single or multiple combination chemotherapy drugs to organoid under different doses. 4) Drug reaction detection: based on integrated sensors, dynamic organoid imaging, activity determination and metabolic product changes are realized to find new drug sensitive markers (such as detection of gene methylation level changes before and after administration). 5) Result interpretation: Based on the observed activity determination and the concomitant biomarkers specially developed for lung cancer PDOs, the drug-molecule docking relationship is established to produce reliable reaction prediction. The test results were evaluated and compared with those of clinical medicine to provide a reference for patient treatment plans.

In a summary, organoids-on-a-chip creates a variety of complex microenvironment components, such as living cells, growth factors, signal gradients *in vivo*, and biological force stimulation by engineering means, with the advantages of high throughput and real-time monitoring by multiple sensors, providing a new idea for personalized drug screening of lung cancer in the future.

## 6 Discussion

The organoids-on-a-chip integrates the advantages of organoid and microfluidic chip technology, and the organoids-on-a-chip is more simulative and controllable, thus it continues to develop towards humanization and automation. In particular, organoids-on-a-chip directly use human tissues and can customize disease models with personalized characteristics according to patient characteristics. It shows great application potential in clinical precision treatment (making personalized treatment plans for patients and searching for candidate markers) and new drug research and development. At present, organoids-on-a-chip companies such as Hesperos, Emulate, Tissuse, Danwang Medical, and Daxiang Science and Technology have sprung up in succession, helping precision medicine and new drug research and development, and realizing the commercial value of organoids-on-a-chip worldwide. However, using microfabricated devices to produce such organoids on a clinically relevant scale is certainly a challenge such as:

(1) there is still a lack of relevant laws, regulations, and management measures for human organs, so it is necessary to study and judge the potential ethical problems caused by the commercialization of human organs and clinical transformation, and formulate corresponding ethical norms and governance measures as soon as possible to realize organoid chips to guide clinical personalized medication.
(2) Organoids-on-a-chip can achieve organoid co-culture or vascularization to a certain extent, but they are still unable to fully reproduce a series of functional changes caused by the complex TME of lung cancer *in vivo.* For example, because lung cancer organs are in a spherical closed state, they are unable to communicate with the external environment if they can be exposed to airborne media. Simulating the interaction between the real-world lung cancer microenvironment and the external environment of viruses, bacteria, and other microorganisms can further explore the mechanisms of microbial metabolism in lung cancer.
(3) In the aspect of manufacturing techniques of microfluidic chips, organoids-on-a-chip models are designed and constructed in a predetermined manner and thus recapitulating dynamic structural, environmental, and functional changes that occur during organogenesis and multiorgan interaction are with complexity. However, As mentioned above, those manufacturing techniques that the main methods used to develop microfluidics devices (e.g. photo-patterning, self-assembly and soft lithography) are cost-ineffective and low-throughput, especially when modeling multiorgan interactions and controlled 3D vascular networks for its bio-and physio-mimetic, the most cutting-edge microfluidic devices for mimicking tumor structure are the 3D printing cancer-on-a-chip models, which provide a multi-organ level dimension with dynamic fluid flow (Neufeld et al., 2022). By combining various biomimetic materials with tumor and stromal cells in a precisely controlled spatial organization, these models can accurately recreate biological structures (Monteiro et al., 2022; Miserocchi et al., 2023).
(4) Integrated with other micro-processing, biosensor, AI, and other technologies to form an automated workstation, it is also necessary to strengthen interdisciplinary cooperation and exchange and establish a closed-loop model for the relevant application of organ technology, including cultivation platforms and testing services, to break the technical barriers and reduce the economic and time costs of laboratory and hospital research.

In conclusion, organoids-on-a-chip is still in the basic research stage. We believe that with the development of manufacturing technology and material science, and the continuous improvement of industry norms, the above problems are expected to be solved. Organoids-on-a-chip will soon become the cornerstone of a new era in precision medicine for lung cancer.
